# Human Intestinal Cells Modulate Conjugational Transfer of Multidrug Resistance Plasmids between Clinical *Escherichia coli* Isolates

**DOI:** 10.1371/journal.pone.0100739

**Published:** 2014-06-23

**Authors:** Ana Manuel Dantas Machado, Morten O. A. Sommer

**Affiliations:** 1 Department of Systems Biology, Technical University of Denmark, Lyngby, Denmark; 2 Novo Nordisk Foundation Center for Biosustainability, Technical University of Denmark, Hørsholm, Denmark; Indian Institute of Science, India

## Abstract

Bacterial conjugation in the human gut microbiota is believed to play a major role in the dissemination of antibiotic resistance genes and virulence plasmids. However, the modulation of bacterial conjugation by the human host remains poorly understood and there is a need for controlled systems to study this process. We established an *in vitro* co-culture system to study the interaction between human intestinal cells and bacteria. We show that the conjugation efficiency of a plasmid encoding an extended spectrum beta-lactamase is reduced when clinical isolates of *Escherichia coli* are co-cultured with human intestinal cells. We show that filtered media from co-cultures contain a factor that reduces conjugation efficiency. Protease treatment of the filtered media eliminates this inhibition of conjugation. This data suggests that a peptide or protein based factor is secreted on the apical side of the intestinal cells exposed to bacteria leading to a two-fold reduction in conjugation efficiency. These results show that human gut epithelial cells can modulate bacterial conjugation and may have relevance to gene exchange in the gut.

## Introduction

The human body is inhabited by a vast number of microorganisms collectively referred to as the microbiota [1]–[3]. The microbiota colonizes every surface of the human body exposed to the environment, including skin, genitourinary, respiratory, and gastrointestinal tracts [3]–[5], with the gastrointestinal tract as the most heavily colonized site in the body [6], [7]. The relationship between the host and its resident microbiota can be mutually beneficial and the microbiota has substantial impact on human health, including dietary and nutritional processing, prevention of pathogen invasion and immune system maturation [8]–[10].

Communication between the human host and its microbiota is necessary for many of these processes. The intestine provides an extensive platform for intercellular signaling between the microbiota, the host, and incoming pathogens. Indeed, intestinal microorganisms secrete molecules that can be sensed by their host, and can also sense host-produced molecules [11], [12]. In addition to such host-microbiota metabolic and signaling interactions, microorganisms also exchange genetic material between them in the gastrointestinal tract. This process of horizontal gene transfer has been implicated in clinical problems with antibiotic resistance [13], [14]. In fact, exchange of antibiotic resistance genes between resistant and susceptible bacteria have been studied in animals and humans [15]–[17].

Horizontal gene transfer can occur through transformation, transduction, and conjugation. It is currently believed that conjugation is the major contributor to the dissemination of antibiotic resistance genes [18]. Conjugation involves the transfer of DNA between cells in a contact-dependent fashion. Plasmids, conjugative transposons, regions of bacterial chromosomes, and integrative and conjugative elements can be transferred via conjugation between remotely related organisms [19]–[23]. While conjugation is recognized to play a key role in the dissemination of antibiotic resistance genes, the influence of the human host on conjugational transfer remains controversial. Several studies have reported inefficient enterobacterial conjugation in intestinal extracts from mice [24] and in the mammalian gut [25], [26]. Yet, other reports identified higher rates of conjugation in the gut [27], [28]. Several factors, including pathogen-driven inflammatory responses occurring in the gut could explain some of these disagreements [29]–[34]. However, there is a need to establish well-controlled model systems in order to improve our understanding of the specific host derived factors that affect bacterial conjugation [35]. In this study we establish such an *in vitro* experimental system using intestinal epithelial cells in co-culture with clinical *E. coli* isolates able to donate and receive an ESBL (extended spectrum beta-lactamase) plasmid. We used this system to determine the impact of human intestinal cells on bacterial conjugation and discovered that an unknown protein or peptide based factor is secreted by intestinal cells reducing the efficiency of bacterial conjugation.

## Materials and Methods

### Cell culture, *E. coli* strains, and growth conditions

Human Caco-2 colorectal adenocarcinoma cells (ECACC 86010202) were grown in transwell filters (Corning) and maintained in Minimal Essential Media (MEM) (Life Technologies) supplemented with 20% fetal bovine serum, 25 µg/mL gentamycin (Sigma), and 0.1 mM non-essential amino acids (Sigma) for 21 days until differentiation occurred. The cell line was maintained at 37°C under 5% CO_2_ humidified atmosphere.

Co-culture was performed using *E. coli* clinical isolates Ec77 and Ec56 (kind gift from Dr. Kristian Schønning, Hvidovre Hospital). Ec77 has an ESBL plasmid and is considered the donor strain. The recipient strain, Ec56, has a kanamycin resistance gene and a gene encoding red fluorescent protein inserted in its Tn7 site. Ec77 and Ec56 were grown in LB supplemented with cefotaxime 2 µg/ml or kanamycin 40 µg/ml, respectively.

### Co-culture of Human Cells

After 21 days of culture, Caco-2 cells were washed three times with phosphate-buffered saline (PBS) 1× and incubated in antibiotic-free medium overnight. *E. coli* colonies were grown overnight and added to the apical side of the intestinal cells at a multiplicity of infection (MOI) of 10 bacteria per cell. Cultures were maintained at 37°C under a 5% CO_2_ humidified atmosphere. Control samples were processed similarly in the absence of intestinal cells. After 2 hours of infection, the media from the apical side of the Caco-2 cells was recovered and plated at the appropriate dilutions in LB plates with cefotaxime 2 µg/ml, kanamycin 40 µg/ml and cefotaxime 2 µg/ml plus kanamycin 40 µg/ml.

### Protease Treatment

Using Caco-2 cells, co-culture was performed as previously described. Media from the apical side was collected, filtered and treated with 2 mg/ml protease (unspecific protease from *Streptomyces griseus*; Sigma) for 10 minutes at room temperature. Treatment with 1∶100 protease inhibitor cocktail (inhibits serine, cysteine, aspartic proteases and aminopeptidases; Sigma) at room temperature followed. *E. coli* strains Ec56 and Ec77 were then cultured in the protease treated media for 2 hours. Control samples were processed similarly in the absence of protease treatment.

### Analysis of Conjugation Efficiency

Conjugation efficiency was calculated in the following manner: number of transconjugants divided by the total number of donor bacteria. Number of transconjugants was calculated by counting the colonies in LB plates with cefotaxime 2 µg/ml plus kanamycin 40 µg/ml. Total number of donor bacteria was calculated by counting the colonies in LB plates with cefotaxime 2 µg/ml.

### Statistical analysis

Conjugation efficiency results were expressed as mean ± SEM of at least three independent experiments and analyzed by Student's *t* test. The differences between data sets were considered significant at *P* values <0.05.

## Results

### Bacterial conjugation efficiency is lower in the presence of intestinal epithelial cells

**Figure 1:**
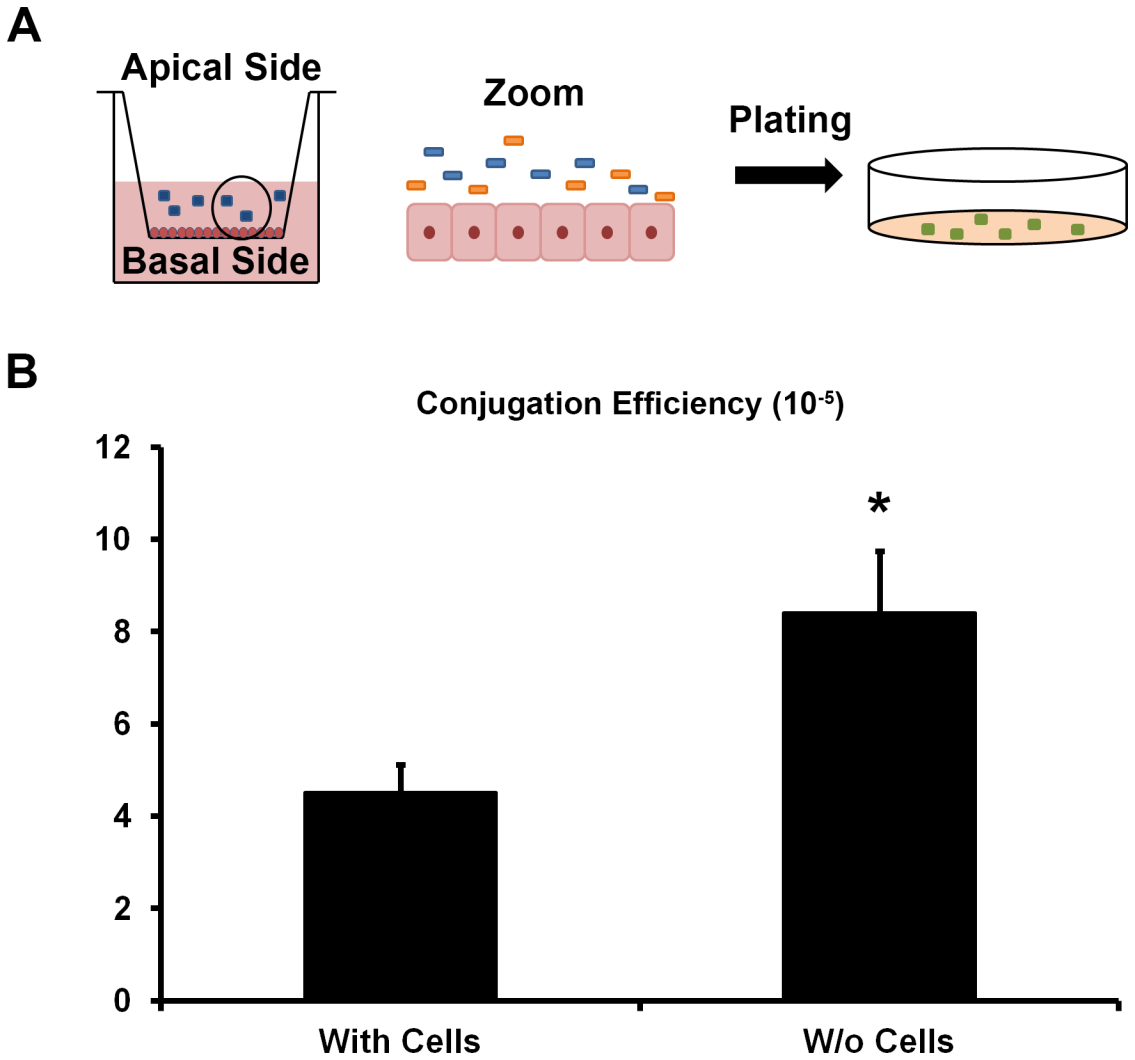
Bacterial conjugation efficiency after co-culture with intestinal cells. (A) Experimental setting. Overview of the setting in a transwell filter and zoom from an area of the filter. In orange and blue are depicted the donor and recipient *E. coli* strains when co-cultured with the intestinal cells without prior treatment. Transconjugants are in green. (B) Efficiency of conjugation after 2 hours of culture of donor and recipient *E. coli* in the presence or absence (w/o) of differentiated intestinal cells, Caco-2. Means ± SEM. Representative of five (with Caco-2 cells) and three (without Caco-2 cells) independent experiments. *, statistically significant from culture with Caco-2 cells (Student's *t* test; *p* = 0.023).

In order to study the potential influence of human intestinal cells on the ability of bacteria to transfer genetic material between them, we used two *E. coli* clinical isolates. The donor strain harbors an ESBL plasmid and the recipient strain has a kanamycin resistance gene and a gene encoding red fluorescent protein inserted in its Tn7 site. The strains were cultured for 2 hours in the presence or absence of differentiated intestinal epithelial cells (Fig. 1A). The intestinal epithelial cells were not exposed to any prior treatment before co-culture with *E. coli*. After this period of co-culture it was observed that conjugation efficiency of bacteria cultured in the presence of the intestinal epithelial cells (4.51×10^−5^) presented a two-fold decrease compared to when cultured in the absence of intestinal cells (8.4×10^−5^; *p* = 0.023) (Fig. 1B). These results show that the presence of intestinal cells decreases the ability of these bacterial strains to perform plasmid conjugation. We recovered a similar number of donor, recipient and transconjugant bacteria after 2 hours in the presence or absence of intestinal cells (Table S1). This observation indicated that the decrease in bacterial conjugation was not due to bacterial killing induced by the intestinal cells.

**Figure 2:**
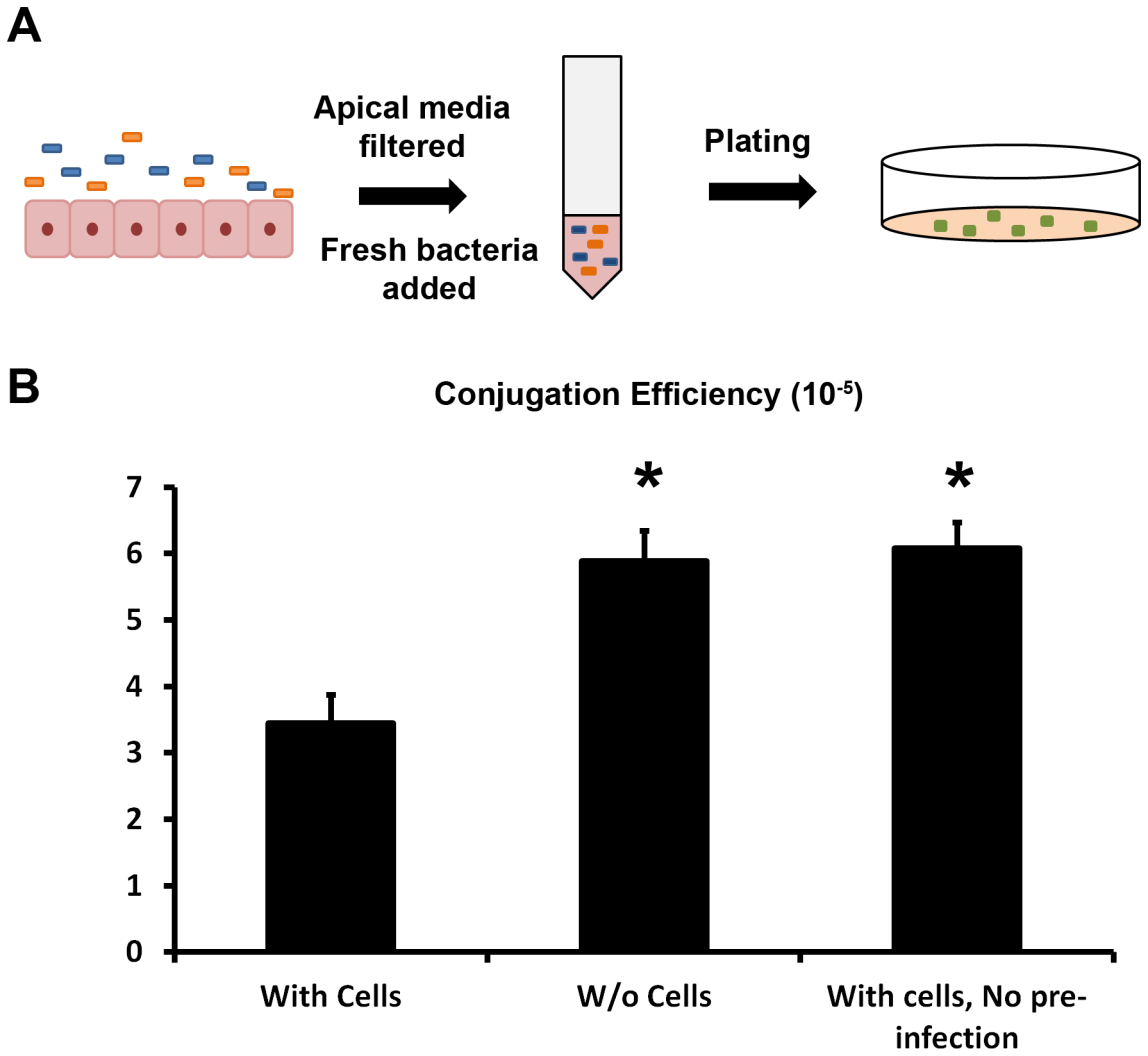
Bacterial conjugation efficiency after culture with media from pre-infected intestinal cells. (A) Experimental setting. In orange and blue are depicted the donor and recipient *E. coli* strains when co-cultured with the intestinal cells and in the filtered media. Transconjugants are in green. (B) Efficiency of conjugation after 2 hours of culture of donor and recipient *E. coli* in culture media that had previously been cultured with or without *E. coli* in the presence or absence (w/o) of differentiated intestinal cells, Caco-2. Means ± SEM. Representative of three (with Caco-2 cells), five (without Caco-2 cells) and four (with Caco-2 cells and without initial pre-infection with *E. coli*) independent experiments. *, statistically significant from culture with pre-infected Caco-2 cells (Student's *t* test; *p* = 0.013, for without Caco-2 cells; *p* = 0.0065, for with Caco-2 cells without pre-infection).

To test whether the reduced conjugation efficiency was dependent on direct contact with the differentiated epithelial cells, we co-cultured *E. coli* donor and recipient strains for 2 hours in the presence or absence of differentiated intestinal epithelial cells. The media from the apical side of the intestinal cells, which represent the intestinal lumen, was recovered and filtered. Fresh donor and recipient strains were co-cultured for 2 hours in the filtered media and the conjugation efficiency was quantified (Fig. 2A). In this set of experiments we also observed a significantly lower conjugation efficiency in the media that had previously been in contact with intestinal cells (3.45×10^−5^) compared to the media that had not been in contact with the intestinal cells (5.89×10^−5^; *p* = 0.013) (Fig. 2B). The efficiency of conjugation in the media that had been in contact with pre-infected intestinal cells was also significantly lower compared to the efficiency of conjugation in the media that had been in contact with intestinal cells where no pre-infection occurred (6.08×10^−5^; *p* = 0.0065) (Fig. 2B). In view of these results we suggest that upon culture with bacteria, intestinal cells secrete an unknown factor that decreases the ability of bacterial cells to perform conjugation.

Similar experiments were performed with the media from the basal side of the intestinal cells. However, no effect was observed on the conjugation efficiency of the bacterial strains (Fig. S1). Therefore we suggest that the unknown factor secreted by the intestinal cells that has an influence on the conjugation efficiency is secreted by the apical side of the intestinal cells.

### Bacterial conjugation is impaired by an unknown peptide or protein secreted by intestinal cells

**Figure 3:**
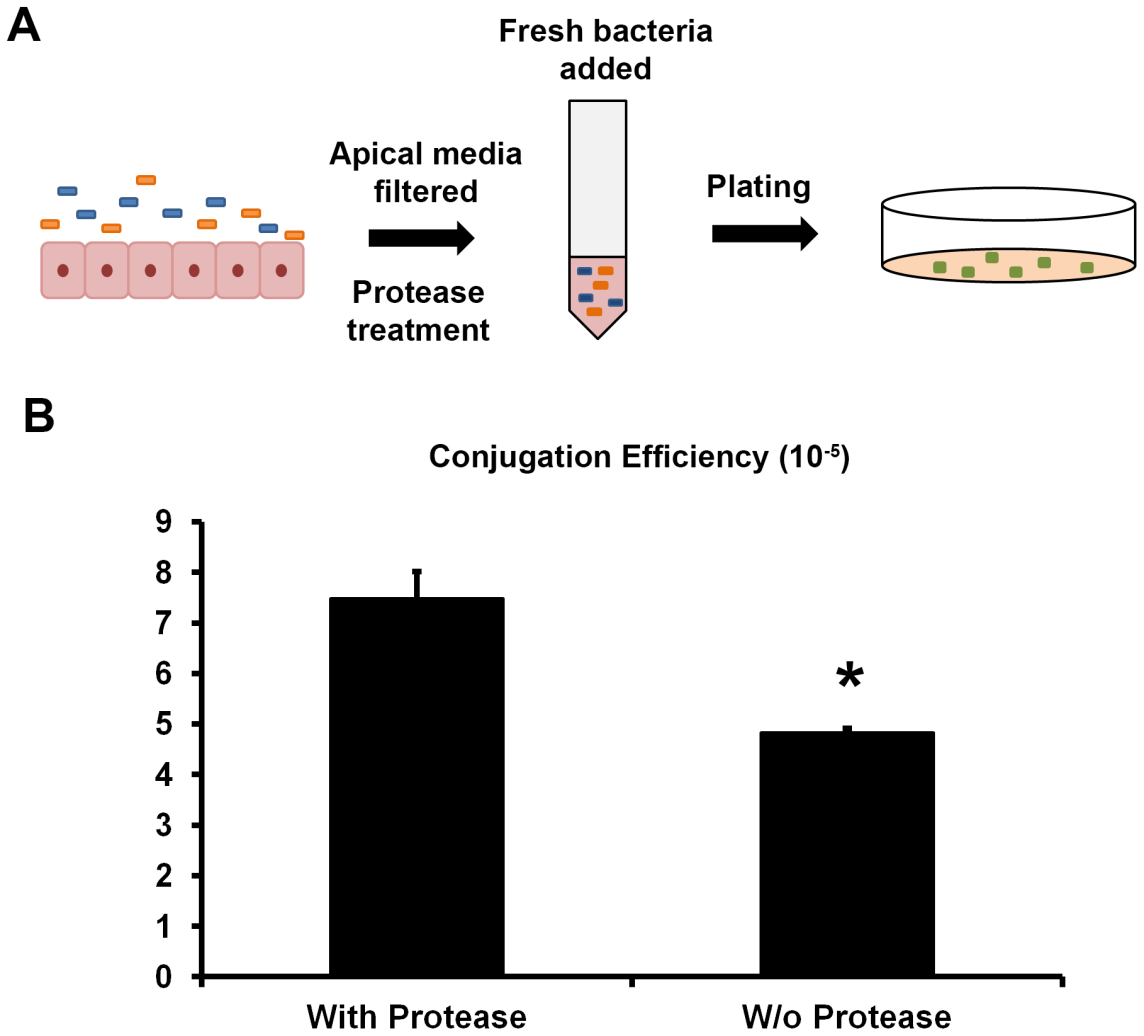
Bacterial conjugation efficiency after culture with protease-treated media from pre-infected intestinal cells. (A) Experimental setting. In orange and blue are depicted the donor and recipient *E. coli* strains when co-cultured with the intestinal cells and in the filtered media. Transconjugants are in green. (B) Frequency of conjugation after 2 hours of culture of donor and recipient *E. coli* in culture media that had previously been cultured with *E. coli* in the presence of differentiated intestinal cells, Caco-2. In this media a protease treatment was applied before the second culture of donor and recipient *E. coli* strains. W/o Protease: without protease treatment. Means ± SEM. Representative of three independent experiments. *, statistically significant from protease treatment (Student's *t* test; *p* = 0.0084).

We wanted to determine if the unknown factor reducing conjugation efficiency, secreted by the intestinal cells when in culture with bacteria, was a protein or peptide based factor. To test this we co-cultured intestinal cells with donor and recipient *E. coli* strains for 2 hours. Media from the apical side of the intestinal cells was recovered, filtered, and treated with an unspecific protease from *Streptomyces griseus*. After treatment, donor and recipient *E. coli* strains were cultured in the media for 2 hours (Fig. 3A). It was observed that in the media that had been treated with protease there was a significantly higher conjugation efficiency (7.48×10^−5^) compared to the media that had not been subjected to the treatment (4.83×10^−5^; *p* = 0.0084) (Fig. 3B). Therefore we suggest that the unknown factor secreted by the intestinal cells which induces lower conjugation efficiency is a peptide or protein, as protease treatment inhibits the effect of the secreted factor.

## Discussion

Bacterial conjugation is considered a major contributor to the dissemination of antibiotic resistance genes in the human gut [18]. Yet, we have a limited understanding of how host factors affect conjugation. We developed an *in vitro* model system that enables controlled investigation of the specific host derived factors that affect bacterial conjugation.

Using this *in vitro* co-culture system we observed that the conjugation efficiency is lowered when clinical *E. coli* isolates are co-cultured with intestinal cells. Our results are in agreement with previous work demonstrating that plasmid transfer between isogenic strains of *E. coli* occurs at a much lower rate in intestinal extracts from mice than in laboratory media [24]. Several other studies report inefficient enterobacterial conjugation in the mammalian gut [25], [26]. Yet, other studies identified higher rates of conjugation in the gut [27], [28], suggesting that poorly understood *in vivo* factors affect transfer of genetic material [29]. For instance, pathogen-driven inflammatory responses occurring in the gut, mediated by the immune system, have been shown to increase *in vivo* conjugation rates, due to a boost in enterobacterial colonization [29]–[34].

In our study, after observing that intestinal cells influence bacterial conjugation efficiency we showed that physical contact between intestinal cells and bacteria is not required for the conjugation process *per se*. Instead it is suggested that an unknown factor is secreted on the apical side of the epithelial cells that decreases bacterial conjugation. Similar examples of such communication and interaction between host and bacteria through secreted, diffusible molecules have been reported [36]–[40]. Finally, we show that protease treatment of the media containing this factor abolishes its inhibitory effect suggesting that the secreted factor is an unknown peptide or protein. Future studies are needed in order to establish the identity of this factor and its relevance *in vivo* as well as to determine the interest of this factor as an adjuvant in antibiotic treatment in order to prevent or decrease the number of antibiotic resistant infections [41].

## Supporting Information

Figure S1 **Bacterial conjugation efficiency after co-culture with basal side of intestinal cells.** Efficiency of conjugation after 2 hours of culture of donor and recipient *E. coli* in the presence or absence (w/o) of differentiated intestinal cells. *E. coli* was co-cultured on the basal side of the intestinal cells. Means ± SEM. Representative of three independent experiments. (Student's *t* test; *p* = 0.987).
(TIF)

Table S1 **Number of donor and recipient** ***E. coli*** **colonies recovered after 2 hours of culture in intestinal cell media.** After 2 hours of culture, the media from the apical side of the Caco-2 cells was recovered and plated at the appropriate dilutions in LB plates with cefotaxime 2 µg/ml and kanamycin 40 µg/ml. Numbers correspond to the average number of colonies obtained after co-culture with intestinal cells (Fig. 1) and after culture with media from pre-infected intestinal cells (Fig. 2). *p* value was calculated using Student's *t* test between the replicates of “with cells” and “without cells” conditions.
(DOCX)
