# Supplementary figures and images for: Human Intestinal Cells Modulate Conjugational Transfer of Multidrug Resistance Plasmids between Clinical Escherichia coli Isolates
Source: PLoS One. 2014 Jun 23;9(6):e100739. doi: 10.1371/journal.pone.0100739 (PMC4067368; doi:10.1371/journal.pone.0100739)

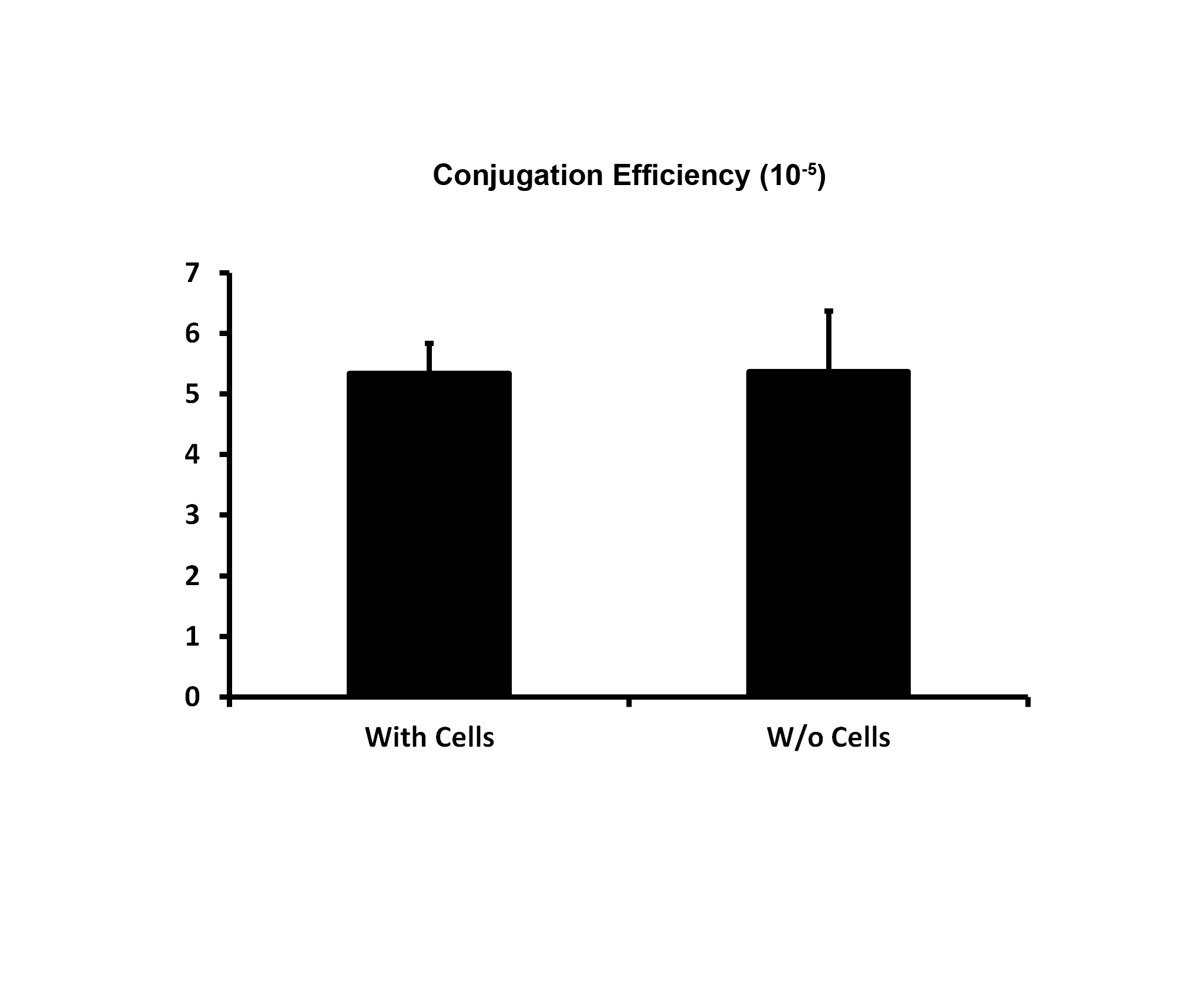

Supplement: Figure S1 — Bacterial conjugation efficiency after co-culture with basal side of intestinal cells. Efficiency of conjugation after 2 hours of culture of donor and recipient E. coli in the presence or absence (w/o) of differentiated intestinal cells. E. coli was co-cultured on the basal side of the intestinal cells. Means ± SEM. Representative of three independent experiments. (Student's t test; p = 0.987). (TIF) [file pone.0100739.s001.tif]
