# Supplementary material for: Human Intestinal Cells Modulate Conjugational Transfer of Multidrug Resistance Plasmids between Clinical Escherichia coli Isolates
Source: PLoS One. 2014 Jun 23;9(6):e100739. doi: 10.1371/journal.pone.0100739 (PMC4067368; doi:10.1371/journal.pone.0100739)
Supplement: Table S1 — Number of donor and recipient E. coli colonies recovered after 2 hours of culture in intestinal cell media. After 2 hours of culture, the media from the apical side of the Caco-2 cells was recovered and plated at the appropriate dilutions in LB plates with cefotaxime 2 µg/ml and kanamycin 40 µg/ml. Numbers correspond to the average number of colonies obtained after co-culture with intestinal cells (Fig. 1) and after culture with media from pre-infected intestinal cells (Fig. 2). p value was calculated using Student's t test between the replicates of “with cells” and “without cells” conditions. (DOCX) [file pone.0100739.s002.docx]

**Table S1**: **Number of donor and recipient *Escherichia coli* colonies recovered after 2 hours of culture in intestinal cell media.**

|  | **With Cells** (10^6^) | | **Without Cells** (10^6^) | | ***p* value** | |
| --- | --- | --- | --- | --- | --- | --- |
|  | Donor | Recipient | Donor | Recipient | Donor | Recipient |
| **Co-culture** | 7 | 7.4 | 5.4 | 7.7 | 0.4021 | 0.8708 |
| **Pre-infection** | 9.6 | 12 | 12 | 15.9 | 0.2695 | 0.0795 |

After 2 hours of culture, the media from the apical side of the Caco-2 cells was recovered and plated at the appropriate dilutions in LB plates with cefotaxime 2 μg/ml and kanamycin 40 μg/ml. Numbers correspond to the average number of colonies obtained after co-culture with intestinal cells (Fig. 1) and after culture with media from pre-infected intestinal cells (Fig. 2). *p* value was calculated using Student’s *t* test between the replicates of “with cells” and “without cells” conditions.
